# Incidence and Patient-Level Risk Factors for Complex Regional Pain Syndrome Following Cubital Tunnel Surgery

**DOI:** 10.1016/j.jhsg.2026.101028

**Published:** 2026-04-24

**Authors:** Stephen J. Perle, Nicholas C. Bank, Sarah Conlon, Alexander D. Jeffs, Stephen M. Himmelberg, J. Megan M. Patterson, Reid W. Draeger

**Affiliations:** ∗ The University of North Carolina School of Medicine, Chapel Hill, NC; † Department of Orthopaedics, The University of North Carolina School of Medicine, Chapel Hill, NC

**Keywords:** Complex regional pain syndrome, Cubital tunnel surgery, Cubital tunnel syndrome, Risk factors, Ulnar nerve decompression

## Abstract

**Purpose:**

Complex regional pain syndrome (CRPS) is a debilitating complication reported after upper-extremity surgeries, yet its incidence following isolated cubital tunnel surgery (CuTS) remains undefined. This study aimed to estimate the incidence of CRPS after isolated CuTS and to identify independent patient-level risk factors.

**Methods:**

A retrospective cohort study was conducted using the TriNetX research database. Patients who underwent a single isolated CuTS between 2000 and 2024 were identified. One-year cumulative incidences of CRPS type I and type II were calculated, excluding individuals with additional upper-extremity trauma or surgery within 1 year before or after CuTS. Separately, multivariable Cox proportional hazards models were performed in the overall CuTS population to evaluate associations between pertinent demographic and clinical factors and time to CRPS development, allowing prior upper-extremity injury and procedural history to be assessed as potential predictors.

**Results:**

The query identified 9,719 patients who met inclusion criteria. At 1 year after surgery, the incidences of CRPS type I and type II were 0.20% and 0.13%, respectively. Women were associated with significantly increased hazard of CRPS I but not CRPS II (HR: 2.16, 95% CI: 1.47–3.17). Preexisting fibromyalgia and polyneuropathy were independently associated with increased hazards of both CRPS subtypes. Prior upper-extremity nerve injury and prior forearm or wrist surgery were associated with increased risk of CRPS I, whereas previous proximal nerve injury and sedative-, hypnotic-, or anxiolytic-related disorders were associated with increased risk of CRPS II.

**Conclusions:**

CRPS following isolated CuTS is exceedingly rare, with an overall 1-year incidence rate of approximately 0.33%. However, identifiable patient-level risk factors, including female sex, preexisting polyneuropathy and fibromyalgia, selected upper-extremity nerve injuries and surgical procedures, were independently associated with increased risk. These findings provide a uniquely large estimate of CRPS risk following CuTS, offering important insight for preoperative counseling and individualized risk stratification.

**Type of study/level of evidence:**

Prognosis II.

Cubital tunnel syndrome is the second most diagnosed compressive neuropathy of the upper extremity and a frequent cause of hand paresthesia, weakness, and pain in the ulnar nerve distribution.^1^^,^^2^ When symptoms are severe, refractory to conservative management, or indicative of progressive motor deficits, surgical intervention remains the mainstay of treatment.^3^ Surgical treatment is most commonly performed via in situ ulnar nerve decompression or anterior transposition of the ulnar nerve.^4^^,^^5^ Although outcomes after cubital tunnel surgery (CuTS) are generally favorable,^3^ postoperative complications can be detrimental, where modest limitations in dexterity or endurance may impair daily activities and occupational performance.^6^^,^^7^

Among postoperative complications, complex regional pain syndrome (CRPS) is uniquely challenging because it may arise after otherwise uncomplicated surgery and can dominate recovery through persistent, disproportionate pain with autonomic and motor disturbances.^8^^,^^9^ It is characterized by sensory, vasomotor, sudomotor, edematous, and trophic changes that extend beyond the expected postoperative course and are often difficult to distinguish early from routine postoperative pain or stiffness.^10^ Because no single pathognomonic diagnostic test exists, CRPS presents heterogeneously and typically requires multimodal management.^11^ Despite its relatively low frequency, CRPS is a high-impact outcome associated with prolonged disability, increased health care utilization, delayed return to work, and elevated medicolegal risk following upper-extremity surgery, accounting for up to 8.8% of malpractice litigation in hand surgery.^11^, ^12^, ^13^, ^14^ CRPS is classically subdivided into type I, which occurs without a clearly identifiable nerve injury, and type II, which is associated with a definable nerve lesion and causalgia.^15^ Although these subtypes are clinically distinct, the underlying pathophysiology remains incompletely understood, and even with early recognition and treatment, a subset of patients develop persistent pain and long-term functional impairment.^11^^,^^16^

Existing estimates of CRPS incidence are heterogeneous and primarily limited to studies on distal radius fractures and procedures of the hand and wrist.^17^, ^18^, ^19^, ^20^, ^21^, ^22^ Data specific to CuTS are comparatively sparse, with CRPS frequently captured as a secondary outcome within single-institution retrospective reviews and case series.^23^^,^^24^ As a result, surgeons lack contemporary evidence that reliably quantifies CRPS risk after CuTS and identifies which patients may be at greatest risk. Prior work in other upper-extremity contexts has suggested associations between CRPS and demographic characteristics, such as female sex and postmenopausal age,^25^, ^26^, ^27^ medical and psychiatric comorbidities,^28^, ^29^, ^30^ chronic pain syndromes,^31^ and substance use,^32^^,^^33^ but these relationships remain controversial, inconsistently replicated, and incompletely understood.^34^ Whether similar factors influence CRPS risk specifically after CuTS, and the magnitude of any such associations, remain important unanswered questions.

Despite its clinical significance, the incidence of CRPS following isolated cubital tunnel surgery has not been well characterized. Accordingly, the purpose of this study was to estimate the incidence of CRPS following CuTS and to identify patient-level risk factors associated with its development using a large-scale, multi-institutional patient network database. We hypothesized that CRPS following isolated CuTS would be relatively rare and that risk factors previously reported in other surgical contexts, including being a woman, psychological comorbidities, preexisting neuropathic or chronic pain conditions, and prior upper-extremity injury or procedures, would be independently associated with its development.

## Materials and Methods

### Database

This retrospective cohort study used the TriNetX Research Network, a global federated health research platform that integrates deidentified electronic health record data from >100 health care organizations and over 140 million patient records. The network provides access to longitudinal clinical information encompassing patient demographics, diagnoses, medications, procedures, and laboratory values. For this investigation, TriNetX’s US Collaborative Network database was queried on November 10, 2025, to capture all adults who underwent CuTS procedures between July 1, 2004, and July 1, 2024. All data were deidentified prior to analysis in accordance with the Health Insurance Portability and Accountability Act Privacy Rule. As a result, this study was considered exempt from institutional review board oversight at our institution. The study methodology and reporting were guided by the Strengthening the Reporting of Observational Studies in Epidemiology recommendations to promote reproducibility, completeness, and transparency.^35^

### Cohort identification

Eligible patients were identified through electronic health record data using the International Classification of Diseases, Tenth Revision, Clinical Modification (ICD-10-CM) and Current Procedural Terminology (CPT) coding systems. The resultant cohort comprised all adults who underwent primary, unilateral CuTS, identified by CPT 64718 (neuroplasty and/or transposition of the ulnar nerve at the elbow), with or without concurrent flexor–pronator muscle lengthening (CPT 24305), between 2004 and 2024. To isolate outcomes attributable solely to CuTS, patients were excluded from the incidence analysis if they had documented concurrent or subsequent upper-extremity trauma or surgical procedures involving the entire upper extremity (shoulder through the fingers) within 1 year before or 1 year after the index CuTS. Excluded events included ICD-10-CM-coded traumatic injuries as well as CPT-coded upper-extremity surgical and endoscopic procedures, including nerve decompressions and arthroscopic interventions. A complete listing of inclusion and exclusion criteria, along with corresponding diagnostic and procedural codes, is provided in Table 1. The date of the CuTS served as the index event for all time-to-event analyses.

**Table 1:** Inclusion and Exclusion Criteria Used to Define the Isolated CuTS Cohort

Procedural Codes	
Inclusion: CuTS	CPT: 64718 (Neuroplasty and/or transposition; ulnar nerve at elbow) ± CPT: 24305 (Flexor–pronator muscle lengthening)
Prior or concurrent exclusions (±1 year)	
Upper-extremity injury	ICD-10-CM: S40–S49 (Injuries to the shoulder and upper arm); S50–S59 (Injuries to the elbow and forearm); S60–S69 (Injuries to the wrist, hand, and fingers)
Cubital tunnel release	CPT: 64718 (Repeat ulnar nerve neuroplasty/transposition at elbow)
Shoulder procedures	CPT: 1004147 (Surgical procedures on the shoulder); CPT: 29805 (Diagnostic shoulder arthroscopy)
Humerus or elbow procedures	CPT: 1004279 (Surgical procedures on the humerus and elbow); CPT: 29830 (Diagnostic elbow arthroscopy)
Forearm or wrist procedures	CPT: 1004420 (Surgical procedures on the forearm and wrist); CPT: 29840 (Diagnostic wrist arthroscopy); CPT: 29848 (Endoscopic carpal tunnel release)
Hand or finger procedures	CPT: 1004616 (Surgical procedures on the hand and fingers)
Other peripheral nerve decompression or repair	CPT: 1009616 (Neuroplasty, major peripheral nerve, arm or leg, open); 64719 (Ulnar nerve at wrist); 64721 (Median nerve at carpal tunnel); 64708 (Neuroplasty, major peripheral nerve, arm or leg, open—other than specified)

### Primary outcomes

The primary outcomes were the 1-year incidences of CRPS type I and type II following the index CuTS. CRPS type I was defined by ICD-10-CM diagnostic codes for CRPS of the upper limb without definable nerve injury (G90.511, G90.512, G90.519). CRPS type II was defined by ICD-10-CM codes for causalgia of the upper limb, indicating confirmed nerve injury (G56.40, G56.41, G56.42). Incident diagnoses were included if recorded within 365 days of the index procedure.

### Cox proportional hazards modeling

Time-to-event analyses were conducted using multivariable Cox proportional hazards regression to identify independent predictors of CRPS type I and type II within 1 year of CuTS. These risk-factor analyses were performed in the broader CuTS population and were analytically distinct from the isolated cohorts used to estimate incidence, allowing prior upper-extremity injury and procedural history to be assessed as potential risk factors. Separate models were constructed for each CRPS subtype.

Covariates were selected a priori based on clinical relevance and prior literature, and encompassed demographics (age at index, sex, and race/ethnicity), mental health and substance use disorders (depression, anxiety, alcohol-, opioid-, tobacco-, cannabis-, stimulant-, sedative-, and other psychoactive substance-related disorders), metabolic and medical comorbidities (overweight/obesity, osteoporosis, rheumatoid arthritis, fibromyalgia, migraine, asthma, ACE-inhibitor use, and connective tissue and autoimmune disorders), diabetes and diabetes-related neuropathic subtypes, polyneuropathy etiologies, and upper-extremity trauma and surgical history unrelated to the index CuTS. Prior upper-extremity conditions and procedures included nerve injuries, fractures, dislocations/sprains, soft-tissue trauma, amputations, and prior nerve, tendon, or osseous procedures at the shoulder, arm, elbow, forearm, wrist, or hand. A lookback window spanning the entirety of each patients’ record was used to assess the presence of covariates for analysis. Hazard ratios (HRs) were reported with 95% confidence intervals (CIs) to determine statistical significance, with patients censored at last recorded encounter. For statistically significant associations, E-values were calculated to quantify the minimum strength of unmeasured confounding required to explain the observed HRs.

## Results

### Incidence of CRPS after CuTS

After application of all inclusion and exclusion criteria, 9,699 individuals comprised the risk set for CRPS I (37 excluded for prior CRPS I diagnosis) and 9,719 for CRPS II (17 excluded for prior CRPS II diagnosis). Within 12 months of the index CuTS, 0.20% of patients developed CRPS I, while 0.13% developed CRPS II. Overall, the 1-year incidence of CRPS following isolated CuTS was approximately 0.33% (Table 2).

**Table 2:** One-Year Incidence of CRPS Following CuTS

Outcome	Analytic Risk Set, n	1-Year Incidence, n (%)
CRPS I	9,699	19 (0.20)
CRPS II	9,719	13 (0.13)

### Risk factors for CRPS after CuTS

In multivariable Cox proportional hazards models following CuTS, being a woman remained the only consistent demographic predictor, with females demonstrating significantly higher hazards of CRPS I (HR: 2.16, 95% CI: 1.47–3.17) but not CRPS II. Advancing age was modestly protective for CRPS I (HR: 0.98 per year, 95% CI: 0.97–0.99), though age was not independently associated with CRPS II (Table 3).

**Table 3:** Selected Independent Predictors of CRPS Following CuTS

Covariate	HR (CRPS I)	95% CI	*P*	HR (CRPS II)	95% CI	*P*
Age at index (per year)	0.98	0.97–0.99	.002∗	0.99	0.97–1.01	.165
F	2.16	1.47–3.17	.000∗	1.54	0.87–2.73	.135
Fibromyalgia	1.88	1.22–2.90	.004∗	2.18	1.18–4.01	.012∗
Polyneuropathy, unspecified	2.36	1.55–3.59	.000∗	2.15	1.14–4.06	.018∗
Chronic pain disorder	1.26	0.72–2.20	.306	1.38	0.69–2.78	.365
Depressive episode	0.80	0.53–1.19	.261	0.70	0.36–1.35	.284
Anxiety disorder	0.82	0.53–1.28	.382	0.78	0.29–2.11	.623
Sedative/hypnotic/anxiolytic-related disorder	1.62	0.35–7.54	.542	7.24	1.80–29.14	.005∗
Alcohol-related disorder	0.47	0.20–1.11	.084	0.59	0.17–2.06	.406
Opioid-related disorder	0.99	0.47–2.09	.968	0.76	0.23–2.50	.652
Nicotine dependence	1.29	0.87–1.92	.212	0.70	0.36–1.35	.284
Overweight/obesity	0.71	0.48–1.04	.077	0.61	0.34–1.10	.099
Rheumatoid arthritis	0.82	0.41–1.62	.561	1.18	0.48–2.91	.715
Fracture of shoulder and upper arm	1.19	0.64–2.19	.582	1.47	0.58–3.71	.419
Fracture of forearm	1.38	0.75–2.52	.299	0.77	0.31–1.87	.557
Fracture at wrist and hand level	0.54	0.30–0.99	.046∗	0.69	0.30–1.61	.388
Injuries to the wrist, hand, and fingers	1.27	0.80–2.02	.306	1.38	0.69–2.78	.365
Nerve injury at shoulder/upper arm level	1.90	0.75–4.83	.178	3.67	1.00–13.46	.050∗
Nerve injury at forearm level	2.41	1.14–5.13	.022∗	0.96	0.23–3.91	.950
Tendon sheath incision for trigger finger	0.98	0.45–2.15	.956	0.95	0.29–3.10	.929
Carpal tunnel release (endoscopic)	0.79	0.36–1.70	.540	0.77	0.26–2.28	.632
Surgical procedures on humerus/upper arm and elbow	1.17	0.72–1.92	.528	1.64	0.83–3.21	.153
Surgical procedures on the forearm and wrist	2.03	1.32–3.13	.001∗	1.74	0.92–3.29	.091
Surgical procedures on the hand and fingers	0.87	0.48–1.58	.653	0.75	0.32–1.79	.521

*P* values reaching statistical significance (*P* < .05).

Conditions reflecting neuropathic vulnerability demonstrated some of the strongest associations. Unspecified polyneuropathy was independently associated with increased hazards of both CRPS I (HR: 2.36, 95% CI: 1.55–3.59) and CRPS II (HR: 2.15, 95% CI: 1.14–4.06). Fibromyalgia similarly conferred elevated risk for CRPS I (HR: 1.88, 95% CI: 1.22–2.90) and CRPS II (HR: 2.18, 95% CI: 1.18–4.01) (Table 3).

Several upper-extremity injury and surgical factors demonstrated subtype-specific associations. For CRPS I, prior forearm-level nerve injury (HR: 2.41, 95% CI: 1.14–5.13) and a history of forearm or wrist surgery (HR: 2.03, 95% CI: 1.32–3.13) were independently associated with increased risk. In contrast, fracture at the wrist or hand level was associated with a modestly protective effect for CRPS I (HR: 0.54, 95% CI: 0.30–0.99). For CRPS II, prior proximal nerve injury at the shoulder or upper arm level emerged as a significant predictor (HR: 3.67, 95% CI: 1.00–13.46). Additionally, substance use disorders involving sedative, hypnotic, or anxiolytic medications were strongly associated with increased CRPS II risk (HR: 7.24, 95% CI: 1.80–29.14) (Table 3).

Notably, opioid-related disorders, depressive and anxiety disorders, alcohol and nicotine use, obesity, osteoporosis, rheumatoid arthritis, and most other evaluated upper-extremity injury or procedure indicators were not independently associated with CRPS I or II in the final adjusted models (Table 3). The complete list of covariates analyzed using Cox proportional hazards models is provided in Table S1, available online on the Journal’s website at https://www.jhsgo.org.

E-value analyses indicated that all statistically significant associations other than age were at least moderately robust to potential unmeasured confounding, with E-values for point estimates ≥3.1. In contrast, the association between advancing age and CRPS I demonstrated limited robustness, consistent with its modest effect size. All E-values are presented in Table S2, available online on the Journal’s website at https://www.jhsgo.org.

## Discussion

In this large, multi-institutional cohort of almost 10,000 adults undergoing isolated CuTS, we found that the 1-year incidence of clinically coded complex regional pain syndrome was low, with approximately 0.20% of patients developing CRPS type I and 0.13% developing CRPS type II. Taken together, these data suggest that the overall risk of CRPS following isolated CuTS is approximately 0.33%. Although uncommon, CRPS after ulnar nerve decompression at the elbow remains a clinically important complication given its potential to dominate the recovery course and lead to persistent pain and disability. Within this rare-event context, we identified several patient-level factors associated with increased hazards of CRPS, including being a woman, preexisting fibromyalgia, and unspecified polyneuropathy for both subtypes, as well as specific patterns of prior upper-extremity nerve injury and surgery and sedative-, hypnotic-, or anxiolytic-related disorders for CRPS II. In contrast, many factors previously implicated in CRPS, including depressive and anxiety disorders, opioid- and alcohol-related disorders, nicotine use, and rheumatoid arthritis,^36^ were not independently associated with CRPS development after CuTS in adjusted models.

The incidence we observed is broadly consistent with, but toward the lower end of, estimates reported after other upper-extremity procedures. The incidence and prevalence following hand surgery, including CuTS, has not been well defined in the literature. Previous reports have demonstrated extreme variability in the occurrence of CRPS across the spectrum of upper-extremity surgery. Prior studies have suggested, more historically, an incidence range of 22% to 39% following distal radius fracture fixation^37^; however, more recent investigation place the approximation around 13.5%.^38^ Estimates range from 0.3% to 40% following fasciectomy for Dupuytren contracture^19^, ^20^, ^21^ and 0.2% to 5% after carpal tunnel release surgery.^17^^,^^18^ Our finding that CRPS after isolated CuTS occurs in roughly 3 per 1,000 patients may reflect several design features: the restriction to isolated CuTS without concomitant or subsequent upper-extremity trauma or surgery, the use of ICD-10-based diagnostic codes rather than symptom-based criteria, and the heterogeneity of diagnostic and coding practices across institutions. Nonetheless, the present study provides a large, procedure-specific estimate of CRPS risk after CuTS and can help frame informed consent and expectation-setting around this complication. Given the frequency of CuTS,^39^ and the variable clinical course of CRPS, timely recognition and accurate estimation of postoperative CRPS risk are essential to mitigate progression to chronic disease and long-term functional impairment.^11^^,^^12^

Our findings are consistent with prior literature showing that CRPS is more frequently diagnosed in females.^25^, ^26^, ^27^^,^^40^ Specifically, after CuTS, we found women to be associated with a significantly increased hazard of CRPS I (HR: 2.16, 95% CI: 1.47–3.17) but not CRPS II. Within our cohort, men had approximately half the hazard of developing CRPS I compared with women, even after adjusting for comorbidities and prior upper-extremity events. Although the underlying mechanisms remain incompletely understood, animal models of CRPS I have shown sex-specific differences, with female mice demonstrating earlier onset and greater magnitude of mechanical allodynia alongside increased inflammatory and oxidative stress markers.^41^ Further evidence indicates female mice exhibit lower nociceptive thresholds, greater motor dysfunction, and evidence of persistent central sensitization despite resolution of acute pain. These findings have been linked to sex-dependent alterations in spinal NR2B expression and inflammation-driven regulation of X-inactive specific transcript, a pathway implicated in chronic pain susceptibility and poor treatment response in CRPS type I.^41^^,^^42^ The absence of an association between women and CRPS type II in our cohort may reflect important pathophysiologic differences specific to nerve injury-associated CRPS that have not been well studied in isolation.^43^ As CRPS II is defined by the presence of a documented nerve injury, it is possible that its etiology is driven more strongly by nerve-specific factors and baseline neuropathic vulnerability than by sex-based susceptibility.

Older studies have reported an association between CRPS and psychological comorbidities, such as depression, anxiety, emotional instability, and recent psychosocial stressers.^28^, ^29^, ^30^ However, this proposed relationship remains contentious as more recent investigations have failed to reproduce these findings.^34^^,^^44^^,^^45^ Our study agrees with contemporary findings as no association was apparent between CRPS and those with International Classification of Diseases codes defining anxiety or depressive disorders following CuTS. However, the presence of sedative-, hypnotic-, or anxiolytic-related disorders was strongly associated with an increased risk of CRPS II, suggesting patients with medication dependence or poorly controlled preoperative anxiety may represent a higher-risk subgroup. Although, it is important to note that the wide confidence interval reflects small event counts and suggests this estimate may be less stable. One interpretation is that such disorders may serve as markers of severe anxiety, insomnia, or other psychosocial stressors that could modulate pain perception, coping, and postoperative recovery.^46^^,^^47^ Similarly, the lack of independent association with opioid-related disorders, alcohol misuse, or rheumatoid arthritis contrasts with some prior reports^31^, ^32^, ^33^^,^^36^ and suggests that, within an isolated CuTS population adjusted for multiple comorbidities, these factors may be less influential than underlying neuropathic and centralized pain conditions.

Comorbid pain and neurologic conditions emerged as some of the most salient risk factors in this analysis. Unspecified polyneuropathy was associated with more than double the hazard of both CRPS I and CRPS II, and fibromyalgia similarly increased the risk of each subtype by approximately twofold. These conditions may reflect a preexisting state of heightened nociceptive and neuropathic vulnerability, in which surgical insult and postoperative inflammation more readily trigger aberrant pain processing and autonomic dysregulation.^48^^,^^49^ Fibromyalgia, in particular, is characterized by central sensitization and amplified pain responses to peripheral stimuli, features that plausibly intersect with current models of CRPS pathophysiology.^50^ Although fibromyalgia and CRPS are distinct clinical entities, overlapping symptom profiles may contribute to diagnostic overlap in administrative data; however, fibromyalgia may also represent a marker of heightened pain vulnerability that predisposes patients to CRPS following surgical insult. The observed associations support the concept that CRPS risk after CuTS is not solely a function of the local nerve decompression but is also shaped by the broader neurologic and pain sensitivity context in which surgery occurs. For patients with known polyneuropathy or fibromyalgia, surgeons may consider additional preoperative counseling regarding the risk of prolonged or disproportionate pain and might collaborate with pain specialists and therapists early in the postoperative course if atypical pain trajectories emerge.

Our findings related to prior upper-extremity injuries and procedures further highlight the importance of preexisting neural and soft-tissue status. Previous work proposes the pathophysiology of CRPS may represent referred pain secondary to injured peripheral nerves, continued peripheral nerve compression, or cutaneous neuromas.^51^ Following isolated CuTS, we observed that prior nerve injury at the forearm level and broad codes for surgical procedures involving the forearm or wrist were associated with higher hazards of CRPS I, whereas prior nerve injury at the shoulder or upper arm level predicted CRPS II. Notably, prior nerve injury in this context reflects a remote history of injury preceding the index procedure and does not contradict the definition of CRPS I, which requires the absence of a definable nerve injury at the time of diagnosis. These associations may reflect cumulative neural insult, altered afferent input, or local changes in microvascular and soft-tissue environment due to autoimmune dysregulation that render the limb more susceptible to dysregulated pain or inflammatory responses after additional surgical intervention.^52^, ^53^, ^54^ The unexpected finding that prior wrist or hand fracture was modestly protective for CRPS I may reflect selection effects rather than a true biologic reduction in risk. Patients who develop CRPS or disproportionate pain after prior fractures may be less likely to pursue or be offered subsequent elective surgery, resulting in underrepresentation of CRPS-susceptible individuals within an isolated CuTS cohort. Additionally, prior fracture experience may recalibrate pain expectations and coping strategies, leading patients to interpret postoperative symptoms as expected and reducing the likelihood of CRPS diagnosis or coding. This observation warrants cautious interpretation and should not be viewed as evidence that prior fractures are protective against CRPS in general.

Although the TriNetX database enables a large, multi-institutional cohort for estimating CRPS incidence, our findings should be interpreted in light of key limitations inherent to the use of a large electronic health record network. A primary limitation of this study relates to the identification of CRPS using administrative coding data. CRPS diagnoses were ascertained using ICD-10-CM codes, and we were unable to verify whether formal diagnostic frameworks, such as the Budapest criteria, were applied. As a result, misclassification is possible, including both overdiagnosis and underdiagnosis. In particular, CRPS may be underreported in administrative data sets, as diagnosis requires clinical recognition and coding by treating providers. Surgeons may be hesitant to assign a CRPS diagnosis following routine procedures because of diagnostic uncertainty or potential medicolegal implications, which may lead to underestimation of the true incidence.

Additional limitations are inherent to the use of an electronic health record database. CPT coding lacks granularity regarding surgical technique, precluding differentiation between in situ decompression and transposition, which may be associated with differing levels of surgical complexity and tissue dissection. Furthermore, we were unable to assess symptom laterality, severity, or duration, nor evaluate treatment response or functional outcomes. As a result, we could not confirm that CRPS diagnoses occurred in the operated limb, and a subset of cases may have represented contralateral CRPS. Our study design minimized this possibility by excluding patients with concurrent or subsequent upper-extremity trauma or procedures surrounding the index operation. Residual misclassification may persist; however, such misclassification would likely be nondifferential and bias estimates of association toward the null, potentially leading to underestimation rather than overestimation of true associations between assessed risk factors and CRPS. Future prospective studies are needed to better characterize CRPS severity and clinical outcomes following CuTS. Finally, this retrospective observational design is subject to residual confounding and cannot establish causality; the identified associations should be interpreted as risk markers rather than definitive causal factors.

## Conflicts of Interest

No authors have any conflicts of interest to report and no benefits in any form have been received or will be received related directly or indirectly to the subject of this article.
