# Supplementary material for: Incidence and Patient-Level Risk Factors for Complex Regional Pain Syndrome Following Cubital Tunnel Surgery
Source: J Hand Surg Glob Online. 2026 Apr 24;8(4):101028. doi: 10.1016/j.jhsg.2026.101028 (PMC13126497; doi:10.1016/j.jhsg.2026.101028)
Supplement: Supplementary Table 1 [file mmc1.docx]

| **Table S1.** Multivariable Cox Proportional Hazards Models Evaluating Patient-Level Predictors of Complex Regional Pain Syndrome (CRPS) Following Cubital Tunnel Surgery | | | | | | |
| --- | --- | --- | --- | --- | --- | --- |
| **Covariate** | **HR (CRPS I)** | **95% CI** | ***P-value*** | **HR (CRPS II)** | **95% CI** | ***P-value*** |
| Age at index (per year) | **0.98** | **0.97–0.99** | **0.002** | 0.99 | 0.97–1.01 | 0.165 |
| Female sex | **2.16** | **1.47–3.17** | **0.000** | 1.54 | 0.87–2.73 | 0.135 |
|  |  |  |  |  |  |  |
| Depressive episode | 0.80 | 0.53–1.19 | 0.261 | 0.74 | 0.39–1.40 | 0.348 |
| Other anxiety disorders | 0.82 | 0.53–1.28 | 0.382 | 1.33 | 0.71–2.49 | 0.366 |
| Other chronic pain | 1.26 | 0.72–3.01 | 0.290 | 1.54 | 0.53–4.48 | 0.424 |
| Fibromyalgia | **1.88** | **1.22–2.90** | **0.004** | **2.18** | **1.18–4.01** | **0.012** |
| Migraine | 1.18 | 0.76–1.83 | 0.468 | 1.18 | 0.46–2.98 | 0.735 |
|  |  |  |  |  |  |  |
| Alcohol related disorders | 0.47 | 0.20–1.11 | 0.084 | 0.59 | 0.17–2.06 | 0.406 |
| Opioid related disorders | 0.99 | 0.47–2.09 | 0.968 | 0.76 | 0.23–2.50 | 0.652 |
| Cannabis related disorders | 0.80 | 0.30–2.09 | 0.644 | 0.27 | 0.03–2.40 | 0.242 |
| Cocaine related disorders | 0.70 | 0.15–3.24 | 0.649 | 0.78 | 0.08–7.15 | 0.823 |
| Sedative, hypnotic, or anxiolytic related disorders | 1.62 | 0.35–7.54 | 0.542 | **7.24** | **1.80–29.14** | **0.005** |
| Tobacco use | 1.25 | 0.69–2.25 | 0.468 | 0.92 | 0.30–2.85 | 0.884 |
| Nicotine dependence | 1.29 | 0.87–1.92 | 0.212 | 0.70 | 0.36–1.35 | 0.284 |
|  |  |  |  |  |  |  |
| Overweight and obesity | 0.71 | 0.48–1.04 | 0.077 | 0.61 | 0.34–1.10 | 0.099 |
| Age-related osteoporosis without current pathological fracture | 1.05 | 0.57–1.94 | 0.870 | 0.78 | 0.29–2.11 | 0.623 |
| Other rheumatoid arthritis | 0.82 | 0.41–1.62 | 0.561 | 1.18 | 0.48–2.91 | 0.715 |
| Systemic lupus erythematosus | 0.71 | 0.37–1.35 | 0.296 | 0.55 | 0.30–1.00 | 0.052 |
| Other systemic involvement of connective tissue | 0.89 | 0.45–1.78 | 0.745 | 0.72 | 0.16–3.22 | 0.669 |
| Asthma | 0.85 | 0.56–1.28 | 0.433 | 1.18 | 0.46–2.98 | 0.735 |
| ACE inhibitors | 1.14 | 0.76–1.72 | 0.531 | 1.18 | 0.46–2.98 | 0.735 |
|  |  |  |  |  |  |  |
| Polyneuropathy, unspecified | **2.36** | **1.55–3.59** | **0.000** | **2.15** | **1.14–4.06** | **0.018** |
| Cerebral infarction | 1.36 | 0.64–2.89 | 0.428 | 0.79 | 0.18–3.62 | 0.754 |
|  |  |  |  |  |  |  |
| Injuries to the shoulder and upper arm | 0.98 | 0.47–2.05 | 0.960 | 1.40 | 0.54–3.61 | 0.489 |
| Injuries to the elbow and forearm | 1.01 | 0.57–1.78 | 0.980 | 0.87 | 0.36–2.10 | 0.764 |
| Injuries to the wrist, hand and fingers | 1.27 | 0.80–2.02 | 0.306 | 1.38 | 0.69–2.78 | 0.365 |
| Fracture of shoulder and upper arm | 1.19 | 0.64–2.19 | 0.582 | 1.47 | 0.58–3.71 | 0.419 |
| Fracture of forearm | 1.38 | 0.75–2.52 | 0.299 | 0.77 | 0.31–1.87 | 0.557 |
| Fracture at wrist and hand level | **0.55** | **0.30–0.99** | **0.046** | 0.69 | 0.30–1.61 | 0.388 |
| Dislocation and sprain of joints and ligaments of shoulder girdle | 0.88 | 0.44–1.75 | 0.720 | 0.95 | 0.32–2.86 | 0.931 |
| Dislocation and sprain of joints and ligaments of elbow | 1.11 | 0.39–3.20 | 0.841 | 1.82 | 0.54–6.21 | 0.339 |
| Dislocation and sprain of joints and ligaments at wrist and hand level | 1.26 | 0.68–2.35 | 0.460 | 0.86 | 0.31–2.36 | 0.763 |
| Injury of nerves at shoulder and upper arm level | 1.90 | 0.75–4.83 | 0.178 | **3.67** | **1.00–13.46** | **0.050** |
| Injury of nerves at forearm level | **2.41** | **1.14–5.13** | **0.022** | 0.96 | 0.23–3.91 | 0.950 |
| Injury of nerves at wrist and hand level | 0.70 | 0.22–2.16 | 0.531 | 0.78 | 0.17–3.64 | 0.754 |
| Injury of muscle, fascia and tendon at shoulder and upper arm level | 0.96 | 0.44–2.06 | 0.910 | 1.03 | 0.36–2.98 | 0.958 |
| Injury of muscle, fascia and tendon at forearm level | 1.28 | 0.64–2.56 | 0.483 | 1.72 | 0.53–5.60 | 0.373 |
| Injury of muscle, fascia and tendon at wrist and hand level | 1.13 | 0.65–1.96 | 0.665 | 0.85 | 0.36–2.02 | 0.711 |
|  |  |  |  |  |  |  |
| Neuroplasty and/or transposition; ulnar nerve at elbow | 0.82 | 0.52–1.27 | 0.368 | 0.70 | 0.34–1.47 | 0.348 |
| Neuroplasty and/or transposition; ulnar nerve at wrist | 1.47 | 0.72–3.01 | 0.291 | 1.54 | 0.53–4.48 | 0.424 |
| Neuroplasty and/or transposition; median nerve at carpal tunnel | 1.41 | 0.92–2.17 | 0.118 | 1.29 | 0.70–2.40 | 0.417 |
| Neuroplasty, major peripheral nerve, arm or leg, open; other than specified | 1.94 | 0.98–3.84 | 0.058 | 2.17 | 0.81–5.84 | 0.125 |
| Endoscopy, wrist, surgical, with release of transverse carpal ligament | 0.79 | 0.36–1.70 | 0.540 | 0.77 | 0.26–2.28 | 0.632 |
| Tendon sheath incision (for trigger finger) | 0.98 | 0.45–2.15 | 0.956 | 0.95 | 0.29–3.10 | 0.929 |
| Excision of ganglion, wrist | 0.80 | 0.37–1.73 | 0.580 | 0.65 | 0.25–1.64 | 0.356 |
| Arthroplasty, interposition, intercarpal or carpometacarpal joints | 1.10 | 0.53–2.25 | 0.805 | 1.49 | 0.57–3.86 | 0.413 |
| Surgical Procedures on the Shoulder | 0.89 | 0.53–1.47 | 0.642 | 0.70 | 0.37–1.34 | 0.285 |
| Surgical Procedures on the Humerus (Upper Arm) and Elbow | 1.17 | 0.72–1.92 | 0.528 | 1.64 | 0.83–3.21 | 0.153 |
| Surgical Procedures on the Forearm and Wrist | **2.04** | **1.32–3.13** | **0.001** | 1.74 | 0.92–3.29 | 0.091 |
| Surgical Procedures on the Hand and Fingers | 0.87 | 0.48–1.58 | 0.653 | 0.75 | 0.32–1.79 | 0.521 |
|  |  |  |  |  |  |  |
| Hispanic or Latino | 1.02 | 0.63–2.70 | 0.470 | 1.49 | 0.57–3.86 | 0.413 |
| Not Hispanic or Latino | 0.87 | 0.56–1.35 | 0.542 | 0.55 | 0.30–1.00 | 0.052 |
| Asian | 1.24 | 0.27–5.76 | 0.781 | 1.72 | 0.20–15.18 | 0.625 |
| Black or African American | 0.98 | 0.47–2.25 | 0.956 | 1.62 | 0.53–4.96 | 0.394 |
| White | 0.97 | 0.55–2.04 | 0.860 | 1.15 | 0.45–2.93 | 0.770 |

95% CI, 95% confidence interval; ACE, angiotensin-converting enzyme; HR, hazard ratio

P-values reaching statistical significance (p<0.05) are listed in bold.
