# Supplementary material for: Incidence and Patient-Level Risk Factors for Complex Regional Pain Syndrome Following Cubital Tunnel Surgery
Source: J Hand Surg Glob Online. 2026 Apr 24;8(4):101028. doi: 10.1016/j.jhsg.2026.101028 (PMC13126497; doi:10.1016/j.jhsg.2026.101028)
Supplement: Supplementary Table 2 [file mmc2.docx]

| **Table S2.** E-values for Statistically Significant Associations Between Cubital Tunnel Surgery and CRPS | | | | | | |
| --- | --- | --- | --- | --- | --- | --- |
| **Covariate** | **Outcome** | **HR** | **95% CI** | **E-value (HR)** | **E-value (95% CI)*** |  |
| Age at index (per year) | CRPS I | 0.98 | 0.97–0.99 | 1.16 | 1.11 |  |
| Female sex | CRPS I | 2.16 | 1.47–3.17 | 3.74 | 2.30 |  |
| Fibromyalgia | CRPS I | 1.88 | 1.22–2.90 | 3.17 | 1.74 |  |
| Polyneuropathy, unspecified | CRPS I | 2.36 | 1.55–3.59 | 4.15 | 2.47 |  |
| Fracture at wrist and hand level | CRPS I | 0.54 | 0.30–0.99 | 3.11 | 1.11 |  |
| Nerve injury at forearm level | CRPS I | 2.41 | 1.14–5.13 | 4.25 | 1.54 |  |
| Surgical procedures on the forearm and wrist | CRPS I | 2.03 | 1.32–3.13 | 3.48 | 1.97 |  |
| Fibromyalgia | CRPS II | 2.18 | 1.18–4.01 | 3.78 | 1.64 |  |
| Polyneuropathy, unspecified | CRPS II | 2.15 | 1.14–4.06 | 3.72 | 1.54 |  |
| Sedative/hypnotic/anxiolytic-related disorder | CRPS II | 7.24 | 1.80–29.14 | 13.96 | 3.00 |  |
| Nerve injury at shoulder/upper arm level | CRPS II | 3.67 | 1.00–13.46 | 6.80 | 1.00 |  |

95% CI, 95% confidence interval; HR, hazard ratio

*E-value for bound closest to null
